# Healthcare usage and cost for plantar fasciitis: a retrospective observational analysis of the 2010–2018 health insurance review and assessment service national patient sample data

**DOI:** 10.1186/s12913-023-09443-2

**Published:** 2023-05-25

**Authors:** Jaeseo Ahn, Jiyoon Yeo, Sook-Hyun Lee, Yoon Jae Lee, Yeoncheol Park, Bonhyuk Goo, In-Hyuk Ha

**Affiliations:** 1 Jaseng Hospital of Korean Medicine, 536 Gangnam-daero, Gangnam-gu, Seoul, 06110 Republic of Korea; 2 Jaseng Spine and Joint Research Institute, Jaseng Medical Foundation, 3F, 538 Gangnam-daero, Gangnam-gu, Seoul, 06110 Republic of Korea; 3 Department of Acupuncture & Moxibustion, Kyung Hee University Hospital at Gangdong, 892, Dongnam-ro, Gangdong-gu, Seoul, 05278 Republic of Korea

**Keywords:** Plantar fasciitis, Health Insurance Review and Assessment Service, National Patients Sample, Cost of care, Healthcare usage

## Abstract

**Background:**

Plantar fasciitis (PF) is the most common cause of heel pain in adults, and the number of patients and medical expenses are increasing annually. However, studies on this condition are lacking. There is a need to investigate universally administered PF treatment and the associated costs. Therefore we analyzed the South Korean Health Insurance Review and Assessment Service data to investigate the distribution and healthcare usage of patients with PF.

**Methods:**

A cross-sectional retrospective observational design was used in this study. Patients diagnosed with PF (ICD-10 code M72.2) between January 2010 and December 2018 in South Korea, of whom 60,079 had used healthcare at least once, were included in the study. We assessed healthcare usage and cost due to PF, treatment method, and visit route. All statistical analyses were performed with descriptive statistics using SAS 9.4 version.

**Results:**

The number of treated cases of PF and patients with PF was 11,627 cases and 3,571 patients in 2010, respectively, which increased annually to 38,515 cases and 10,125 patients by 2018, respectively. The 45-54-year-old age group had the highest number of patients; the patient population was predominantly women. Physical therapy was used frequently in Western medicine (WM) institutions, where > 50% of the medicines prescribed to outpatients were analgesics. In contrast, acupuncture therapy was most commonly used in Korean medicine (KM) institutions. A high percentage of patients who visited a KM institution, followed by a WM institution, and then returned to the same KM institution had visited the WM institution for radiological diagnostic examination.

**Conclusion:**

This study analyzed nine years of period data from a patient sample of claims data from the Health Insurance Review and Assessment Service to examine the current status of health service use for PF in Korea. We obtained information on the status of WM/KM institution visits for PF treatment, which could be useful for health policymakers. Study findings regarding treatments often used in WM/KM, the frequency of treatments, and their costs could be used as basic data by clinicians and researchers.

**Supplementary Information:**

The online version contains supplementary material available at 10.1186/s12913-023-09443-2.

## Background

Plantar fasciitis (PF) is the most common cause of heel pain among adults, affecting 10% of the US population. In the US, over 600,000 outpatients visit a medical institution annually for this condition [1]. Furthermore, PF can affect anyone, regardless of lifestyle [2]. Heel pain has been reported in 7% of people aged ≥ 65 years, and it is estimated that it causes over one million people in the US to visit a physician per year [3–5]. The physician visit rate for PF is 8.2 per 1,000 among individuals aged 45–64 years, which is markedly higher than the rate among those aged 25–44 years [6]. According to a recent study, the annual cost associated with PF is $284 million, while the cost per visit was found to be approximately $50 and the average cost of NSAIDs (generic first-line therapy) was $600 per person per year [7]. According to the Korean National Health Insurance Statistical Yearbook, the number of patients treated for plantar fascial fibromatosis and fasciitis (ICD-10 code M72.2) increased from 89,906 to 2010 to 259,104 in 2018, while the treatment cost has increased from 5.6 billion to 19.1 billion won (Korean currency) for the same period [8].

The plantar fascia is a connective tissue spanning from the calcaneus bone to the tendons of the forefoot and proximal phalanx. It is responsible for shock absorption and supports the arch of the foot [7, 9]. The exact cause of PF is uncertain; however, abnormal biomechanics are considered possible causes [7, 10]. The progression of PF involves degeneration and inflammation caused by micro-injuries in the bone-tendon junction of the calcaneus bone [5]. PF generally causes heel pain, [1] which is exacerbated by walking, exercising on a firm surface, or standing for long periods [3, 4]. The risk of PF is higher among women, middle-aged patients, long-distance runners, soldiers, obese patients, and patients with either pes cavus (high-arched) or pes planus (flat) foot deformity [3, 4].

Regarding the treatment of PF, 90% of patients undergo conservative treatments including rest, non-steroidal anti-inflammatory drugs (NSAIDs), stretching, foot bracing, corticosteroid injection, extracorporeal shock wave therapy, and ultrasound therapy [4]. The use of analgesics is the most common treatment, accounting for 47% of all treatments, while exercise counseling and physical therapy account for 26% and 19%, respectively [6]. If heel pain is not resolved within 12 months of conservative treatment, then surgery may be indicated [11, 12].

Following the increasing number of cases and costs related to PF, it has become necessary to establish guidelines [2, 6] and treatment methods [13] at the national level. Thus, there is a clear clinical need to investigate the treatment universally administered for PF and the associated costs. The Korean medical system is a dual system in which both KM and WM, based on biomedical medicine, coexist. KM originated in Korea and has been continuously developed as a traditional medicine. Composed mainly of acupuncture and herbal medicine, KM treatments include meditation and qigong, massage, and bone setting. WM was introduced in Korea at the end of the 19th century and has supplanted KM in terms of health care [14]. Because of a dual healthcare system—WM and KM—, patients may select their preferred type of medical institution for a disease. This study was conducted to present primary evidence for providing the status of patients’ visits to medical institutions and medical costs and for improving policies related to the problems of the dual healthcare system. Therefore, this study aimed to analyze the status, treatment details, and visit route of patients with PF using the claims data of the Health Insurance Review and Assessment Service and provide primary evidence regarding the status of medical use and cost of PF for experts such as health policymakers, clinicians, and researchers.

## Methods

### Data source

This study used the Korea Health Insurance Review and Assessment Patient Sample (HIRA-NPS), representing approximately 3% of samples extracted from the total patient population of a given year over nine years between 2010 and 2018 [15]. Claims data are collected through claims of the entire population from medical institutions for the medical care benefit review and evaluation of the appropriateness of medical care benefits by the National Health Insurance Act of the Republic of Korea. The claims data includes qualifications and insurance premiums of all citizens, health checkup results, and medical treatment details. Additionally, health insurance claims data are generated when a medical facility files a claim for an insurance payment to the HIRA after providing a patient medical service(s). Data collected for purposes other than research are called secondary data. Secondary data statistically sampled after removing personal and business information from the raw data were used. The data are supplied according to the stratified systematic sampling of patient units according to gender and age groups (10-year units) and include patient treatment and prescription details for one year from the start date of care for the given year [16].

### Study design and population

**Fig. 1:**
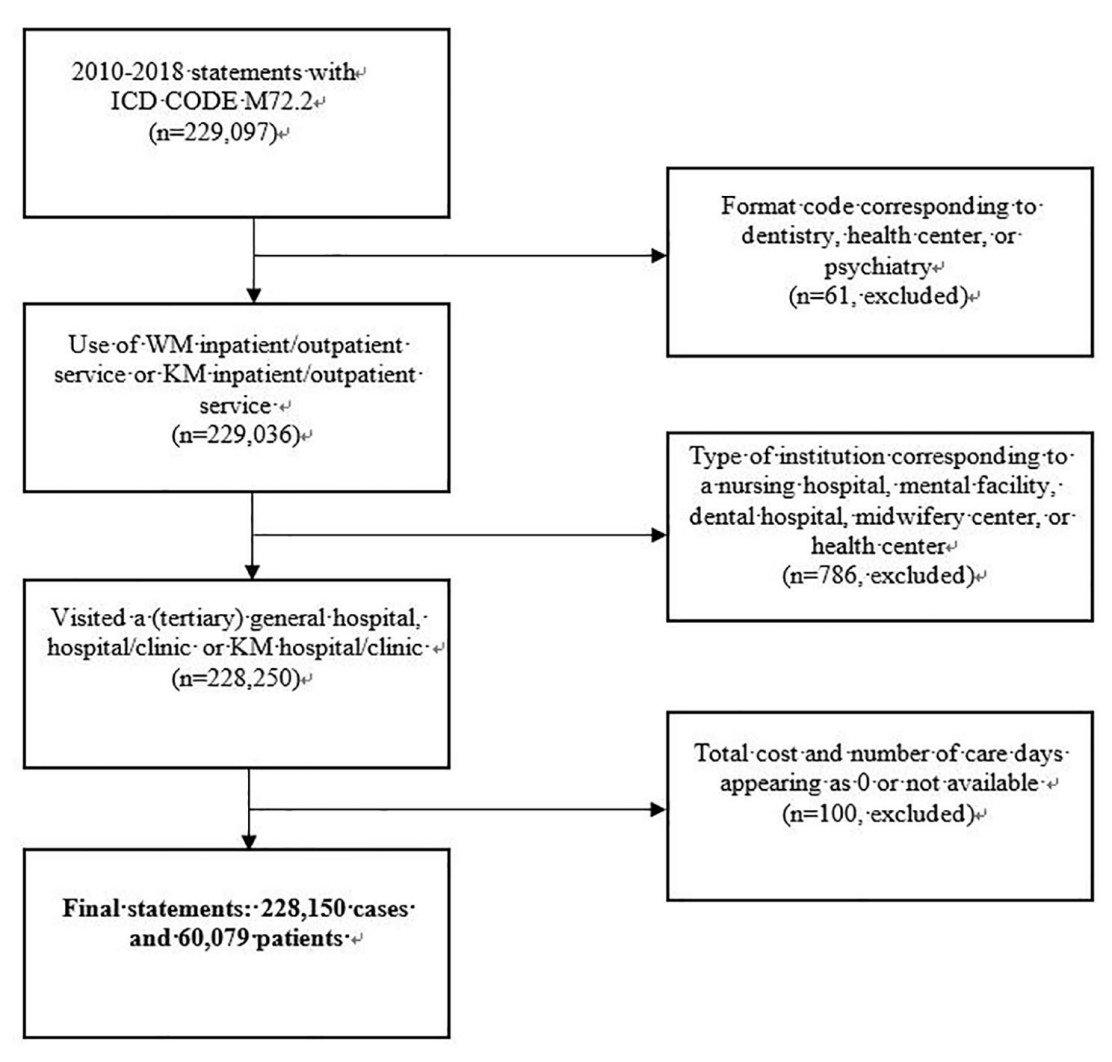
Flowchart of the study This flowchart shows the inclusion and exclusion of participants in the study from the 2009–2018 Korea Health Insurance Review and Assessment Patient Sample

A cross-sectional design was used for this study. The study population consisted of patients of all ages treated at least once, with ICD-10 M722 (PF) as the primary diagnosis code between 2010 and 2018 in South Korea. In total, 228,150 treatment records of 60,079 patients were analyzed after excluding the following cases: format code corresponding to dentistry, health center, or psychiatry (n = 61); type of institution corresponding to nursing hospital, mental facility, dental hospital, midwifery center, or health center (n = 786) (Fig. 1).

### Data analysis

Patients with PF were classified according to age (eight groups in 10-year increments, from < 15 to ≥ 75 years), gender, payer type (health insurance, Medicaid, and others), type of visit (inpatient and outpatient), and type of medical institution (tertiary hospital/general hospital/hospital, clinic, KM hospital, and KM clinic). Frequency analysis was performed for each classification.

To investigate the changes in treatment for PF that have taken place over nine years, the average expenditure and the average number of treatments were tallied for each item. The log change of the annual average of each item (consultation fee, injection fee, physical therapy, imaging diagnosis, treatment/surgery fee, examination fee, hospitalization fee, medication fee, etc.) in 9 years, and differences between KM and WM were analyzed.

**Fig. 2:**
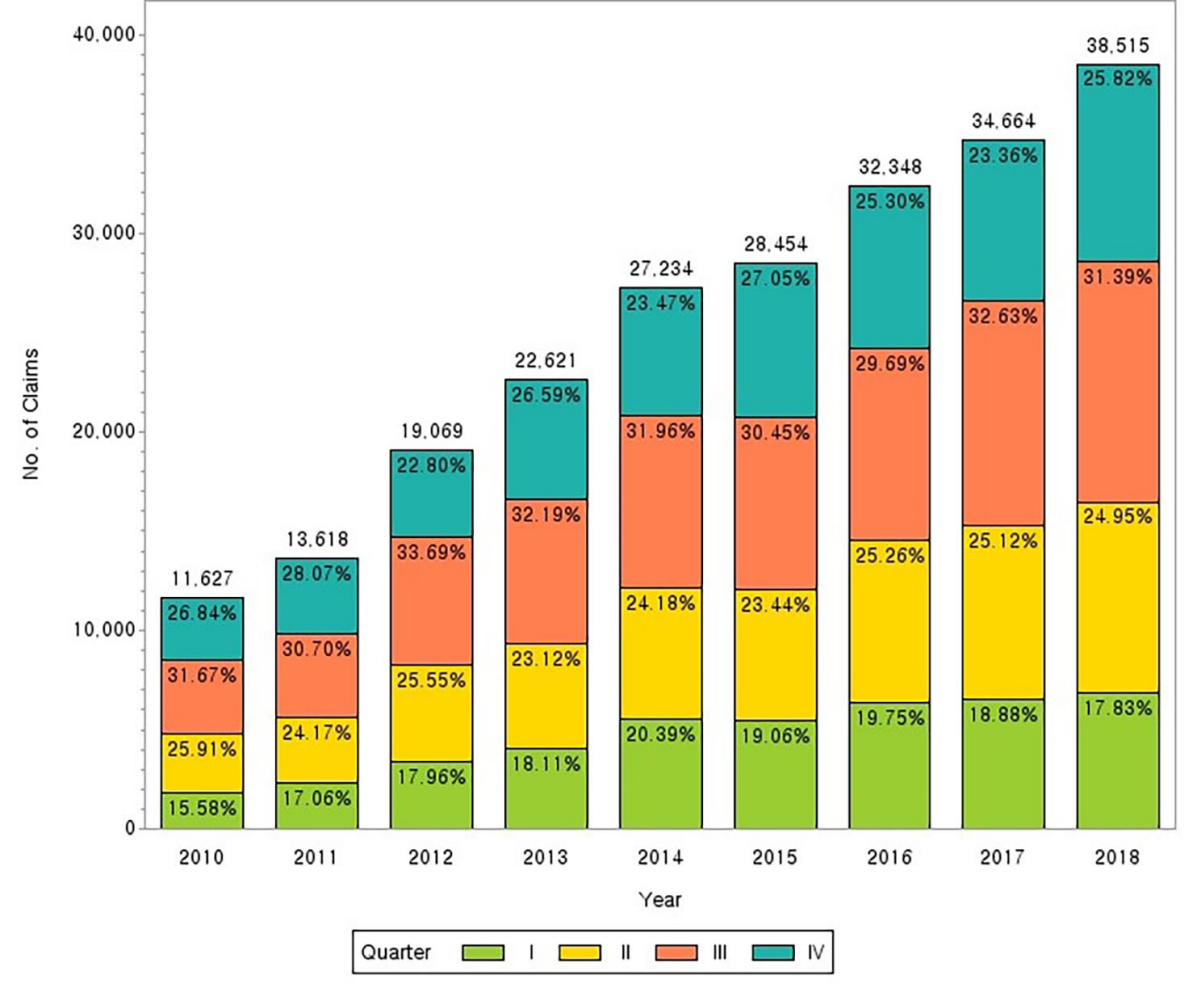
Quarterly number of plantar fasciitis treatment cases QI: January, February, and March; QII: April, May, and June; QIII: July, August, and September; and QIV: October, November, and December

The route of a hospital visit by patients with PF was classified into six types; (1) visited only Korean medical institutions more than once a year; (2) after using Korean medical institutions, transferred to western medical institutions and did not return to Korean medical institutions; (3) repeated the pattern of alternately using Korean medical institutions, Western medical institutions, and Korean medical institutions one or more times; (4) visited only Western medical institutions more than once a year; (5) after using Western medical institutions, transferred to Korean medical institutions and did not return to Western medical institutions; (6) repeated the pattern of alternately using Western medical institutions, Korean medical institutions - Western medical institutions one or more times. The frequency and percentage were analyzed. Lastly, to determine whether there are seasonal variations in the incidence of PF, treatment cases for PF were analyzed yearly and quarterly (Fig. 2).

All costs were converted using the average the Korean Republic won - United States dollar (KRW-USD) exchange rate for a specific year and were adjusted based on the healthcare consumer price index (see Table S1). All statistical analyses were performed using SAS 9.4 TS Level 1M4 (2002–2012, SAS Institute Inc., Cary, NC, USA).

### Patient and public involvement

Patients or the public were not involved in this work.

## Results

This study included 60,079 patients and their 228,150 medical records with PF, whose KCD-10 code for primary diagnosis was M722, for nine years from 2010 to 2018.

### Characteristics of patients with PF

More women (57.71%) than men (42.29%) were treated for PF (Table 1), and patients between the ages of 45–54 years were most common (26.06%). When comparing healthcare utilization by gender, more women visited medical institutions than men (57.71%). The treatments for PF were dominantly outpatient (99.88%, Table S2), and the type of WM institution was largely primary care clinics (75.16%). The type of KM institutions visited to treat PF were also primary care clinics (97.65%).

**Table 1:** Patient characteristics

Category		Patient							
		Total (2010–2018)		Visited a Western medicine institution only (2010–2018)		Visited a Korean medicine institution only (2010–2018)		Visited both institutions (2010–2018)	
		n	%	n	%	n	%	n	**%**
Age (years)	< 15	1948	3.24	1659	3.67	272	2.34	17	0.53
	15–24	4059	6.76	3358	7.42	608	5.22	93	2.92
	25–34	7645	12.72	6044	13.36	1302	11.18	299	9.39
	35–44	11,740	19.54	9129	20.18	2031	17.44	580	18.21
	45–54	15,654	26.06	11,690	25.84	2989	25.66	975	30.61
	55–64	11,611	19.33	8402	18.57	2436	20.91	773	24.27
	65–74	5710	9.5	3836	8.48	1505	12.92	369	11.59
	≥ 75	1712	2.85	1128	2.49	505	4.34	79	2.48
Gender	Men	25,405	42.29	19,420	42.92	4789	41.11	1196	37.55
	Women	34,674	57.71	25,826	57.08	6859	58.89	1989	62.45
Payer type	National Health Insurance *	58,548	97.45	44,103	97.47	11,325	97.23	3120	97.96
	Medicaid	1503	2.5	1115	2.46	323	2.77	65	2.04
	Others	28	0.05	28	0.06	-	-	-	-

### Average rate of change in total expenditure and number of claims according to each item

The highest expenditure for PF among the types of treatments was injection (9-year total expenditure average: $101,548) with an average annual increase of 20%, and physical therapy (9-year total expenditure average: $57,387) with an average annual increase of 11.87% (Table S3). For WM treatment alone, physical therapy ($57,387) and diagnostic radiology ($48,364) accounted for the highest expenditure to treat PF aside from examination fees. Regarding the expenditure only from the Western medical institutions, the ranking of the expenditure was in the order of examination fee ($172,806), physical therapy ($57,387), diagnostic radiology ($48,364), treatment/surgery fee ($17,939), and injection fee ($10,847). The item with the highest average increase rate over the nine years was the treatment/surgical fee, which increased by 18.71% annually. The highest expenditure for KM treatment was acupuncture ($90,701). The item with the highest average rate of annual increase was medication (9-year total expenditure average: 39.91% per year).

### Route of a hospital visit

The 45–54 age group was the highest group of patients with hospital visits to treat PF (Table 2). There was a decreasing trend for those of younger and older age. The group with the most patients comprised women aged 45–54, and the groups that only visited KM institutions or WM institutions showed a similarly high percentage of patients aged 45–54. (1) Visited a KM institution only at least once per year 25.66%; (2) Visited a KM institution, switched to a WM institution, and did not return to the KM 28.57%; (3) Repeated the alternating pattern of ‘KM -WM- KM at least once 26.04%; (4) Visited a WM institution only at least once per year 25.84%; (5) Visited a WM institution, switched to a KM institution, and did not return to the WM institution 32.76%; (6) Repeated the alternating pattern of WM - KM - WM at least once 0.38%.

**Table 2:** Route of hospital visits according to age and gender

Group	Age	Total		Sex			
				Male		Female	
		n	%	n	%	n	**%**
(1) Visited a KM institution only at least once per year	Under 15	272	2.34	181	1.55	91	0.78
	15–24	608	5.22	308	2.64	300	2.58
	25–34	1302	11.18	743	6.38	559	4.80
	35–44	2031	17.44	979	8.40	1052	9.03
	45–54	2989	25.66	1056	9.07	1933	16.60
	55–64	2436	20.91	821	7.05	1615	13.87
	65–74	1505	12.92	517	4.44	988	8.48
	75 or older	505	4.34	184	1.58	321	2.76
	Subtotal	11,648	100.00	4789	41.11	6859	58.89
(2) Visited a KM institution, switched to a WM institution, and did not return to the KM institution	Under 15	1	0.10	1	0.10	-	-
	15–24	27	2.76	17	1.73	10	1.02
	25–34	94	9.59	59	6.02	35	3.57
	35–44	167	17.04	89	9.08	78	7.96
	45–54	280	28.57	88	8.98	192	19.59
	55–64	259	26.43	85	8.67	174	17.76
	65–74	117	11.94	39	3.98	78	7.96
	75 or older	35	3.57	13	1.33	22	2.24
	Subtotal	980	100.00	391	39.90	589	60.10
(3) Repeated the alternating pattern of ‘KM -WM- KM at least once	Under 15	-	-	-	-	-	-
	15–24	10	2.96	8	2.37	2	0.59
	25–34	29	8.58	16	4.73	13	3.85
	35–44	66	19.53	39	11.54	27	7.99
	45–54	88	26.04	30	8.88	58	17.16
	55–64	86	25.44	27	7.99	59	17.46
	65–74	49	14.50	11	3.25	38	11.24
	75 or older	10	2.96	3	0.89	7	2.07
	Subtotal	338	100.00	134	39.64	204	60.36
(4) Visited a WM institution only at least once per year	Under 15	1659	3.67	1024	2.26	635	1.40
	15–24	3358	7.42	1708	3.77	1650	3.65
	25–34	6044	13.36	3273	7.23	2771	6.12
	35–44	9129	20.18	4308	9.52	4821	10.66
	45–54	11,690	25.84	3930	8.69	7760	17.15
	55–64	8402	18.57	3012	6.66	5390	11.91
	65–74	3836	8.48	1629	3.60	2207	4.88
	75 or older	1128	2.49	536	1.18	592	1.31
	Subtotal	45,246	100.00	19,420	42.92	25,826	57.08
(5) Visited a WM institution, switched to a KM institution, and did not return to the WM institution	Under 15	13	1.12	12	1.03	1	0.09
	15–24	38	3.28	18	1.55	20	1.72
	25–34	130	11.21	68	5.86	62	5.34
	35–44	213	18.36	98	8.45	115	9.91
	45–54	380	32.76	98	8.45	282	24.31
	55–64	237	20.43	71	6.12	166	14.31
	65–74	128	11.03	47	4.05	81	6.98
	75 or older	21	1.81	9	0.78	12	1.03
	Subtotal	1160	100.00	421	36.29	739	63.71
(6) Repeated the alternating pattern of WM - KM - WM at least once	Under 15	3	0.00	2	0.00	1	0.00
	15–24	18	0.03	13	0.02	5	0.01
	25–34	46	0.08	23	0.04	23	0.04
	35–44	134	0.22	62	0.10	72	0.12
	45–54	227	0.38	59	0.10	168	0.28
	55–64	191	0.32	57	0.09	134	0.22
	65–74	75	0.12	28	0.05	47	0.08
	75 or older	13	0.02	6	0.01	7	0.01
	Subtotal	707	1.18	250	0.42	457	0.76
Total		60,079	100.00	25,405	42.29	34,674	57.71

Only the group that visited WM institutions had a higher percentage of younger patients than the group that only visited KM institutions, whereas the group that only visited KM institutions had a higher percentage of older patients than the group that only visited WM institutions. Men visited medical institutions for PF more frequently than women of younger ages, while the proportion of women constantly rose with age.

### Percentage of WM institution visits among patients with repeated visits to KM and WM

Among the patients who visited both KM and WM institutions to treat PF, those who were first treated in a KM institution and made following visits to a WM institution received physical therapy (44.93%), examination (36.03%), and diagnostic radiology (7.85%) before receiving treatments again in a KM institution. The proportion of patients who received their first diagnosis at a Korean medical institution and returned to the Korean medical institution within seven days thereafter was 13.58%, which was nearly double the original proportion of 7.85%. More than 15.5% of those who received diagnostic radiology services repeated the alternating pattern of ‘WM-KM’ at least once and returned to their first KM institution within a week (Table 3).

**Table 3:** Middle treatment received among patients switching back and forth between Korean Medicine and Western medicine

Category	Repeated the alternating pattern of Korean medicine –Western medicine- Korean medicine at least once in total (a)		Returned within 7 days (b)		(b/a)	Returned within 14 days (c)		(c/a)
	n	%	n	%	%	n	%	%
Physical therapy	2620	44.93	237	45.32	9.05	398	47.21	15.19
Examination	2101	36.03	168	32.12	8.00	270	32.03	12.85
Diagnostic radiology	458	7.85	71	13.58	15.50	105	12.46	22.93
Injection*	264	4.53	36	6.88	13.64	55	6.52	20.83
Testing	250	4.29	0	0.00	0.00	0	0.00	0.00
Anesthesia	77	1.32	2	0.38	2.60	5	0.59	6.49
Others	61	1.05	9	1.72	14.75	10	1.19	16.39
Total	5831	100.00	523	100.00	8.97	843	100.00	14.46

*Injections: For Korean Medicine, reimbursements under “Injection” include acupuncture, moxibustion, and cupping

### Quarterly number of treatment cases

To check for seasonal variations in the incidence of PF, each year was divided into quarters (QI: January, February, and March; QII: April, May, and June; QIII: July, August, and September; and QIV: October, November, and December), and the number of cases was analyzed annually and quarterly (Fig. 2). The number of treatment cases for PF showed a distinct increasing trend. In each year, the highest percentage of cases for PF was observed at QIII, at approximately 30%. The number of treatment cases in QI was the lowest.

### Use of anti-inflammatory agents

Anti-inflammatory agents were the most commonly prescribed drugs for outpatients of WM institutions, followed by gastrointestinal drugs and muscle relaxants. The number of drug prescriptions increased annually (Figure S1). When anti-inflammatory agents were divided into different types, the most commonly prescribed anti-inflammatory agents were NSAIDs, anti-inflammatory enzymes, and antiplatelet agents (Table S4). The prescription of each of these drugs also increased yearly (Table S4 and Figure S2).

## Discussion

This study analyzed the status, treatment details, and visit routes for PF in Western and Korean medical institutions using the HIRA-NPS data for a total of 9 years from 2010 to 2018. The results found that the number of patients with PF, the number of treated PF cases, and the total amount of medical costs have been increasing every year, both in Western and Korean medical institutions. The comparison of the number of patients between the Western and Korean medical institutions showed Western medical institutions to have three to four times as many patients than medical institutions every year, and the annual fluctuation was not large.

Only 12% of inpatients and 39% of both inpatients and outpatients received drug prescriptions, showing that drug prescription was less common in Korea compared to the US, where drugs were reported to be prescribed in 46.6% of cases [2, 6]. A recent finding that NSAIDs alone were not effective for patients with PF may explain the finding of fewer drug prescriptions [13].

With respect to KM, the annual average expenditure was the highest for acupuncture, moxibustion, and cupping. As shown in Table S5, most patients received acupuncture therapy from KM institutions. According to previous studies, acupuncture is effective in reducing pain and has few adverse effects [17, 18]. The average cost per acupuncture treatment is $3.74, but the average per-patient cost is $39.16, indicating that patients received an average of approximately 10.47 rounds of acupuncture for PF. Furthermore, previous studies show that dry cupping therapy is effective in reducing pain [19]. In addition, the results indicate that the rate of herbal medicine prescriptions is increasing each year [20].

Plantar injuries have been reported to become more severe after the age of 40 years due to reduced shock absorption by the fat pads on the soles of the feet [13]. The highest incidence was found in women aged 45–54 years in this study, as has also been shown in previous studies [2, 4, 10].

Women tend to make up the larger proportion of patients with PF [4, 10, 21]. In this study, when patients were examined according to age groups, there were more men than women with PF up to the age of 35 years; however, beyond this age, women were more often treated for PF. Considering that physically burdensome activities could result in PF, [2, 4–7, 13] these results may be explained by men being more physically active in this regard than women at a younger age [6, 22]. In the same context, previous studies have reported that the feet are the most often injured among foot soldiers, [23] and soldiers have a high incidence of PF [4, 24]. This coincides with the Korean conscription system, where a 2-year military service is mandatory for men aged 19–34 years.

In this study, the percentage of affected women increased with age. While no studies have identified the reason for this, previous studies have reported that intrinsic muscle weakness is a risk factor [24, 25] and that women have less skeletal muscle mass than men in older age, [26] which may contribute to the increasing percentage of women with PF with advanced age. Among patients who visited medical institutions for PF, the number of women was approximately 1.36 times higher than the number of men [4, 10, 21]. The prevalence by age group was the highest in those aged 45–54 years in both WM and KM clinics, and older age groups showed a relatively higher preference for KM treatments [27]. Women preferred KM clinics over Western medicine clinics for musculoskeletal disorders compared to men [27].

Furthermore, the healthcare utilization of patients with PF showed a drawback of the dual healthcare system in Korea, where patients had to visit WM institutions for radiological examination to confirm the diagnosis, only to be treated again at KM institutions. With an increasing number of patients with musculoskeletal disease, this may cause inconveniences on the part of healthcare users and unnecessary medical expenditures. On the other hand, diagnostic radiology and physical therapies can only be claimed by WM in Korea. As shown in Table S5, radiological examination showed the largest annual rate of growth besides surgery in terms of total expenditure, despite recent findings advising against indiscriminate radiological examinations for heel pain unless a fracture is suspected [4, 25].

## Limitations and strengths

Our study has several limitations. We used the data of medical claims in Korea related to PF. However, it was difficult to determine whether patients received treatment for only PF. To overcome this, only patients with PF as the main diagnosis were included. However, treatments for PF and other diseases could not be completely separated.

Second, the data were annual and not continuous. Consequently, patient follow-up was possible within the same year but no longer once the year changed; therefore, previous history could not be determined.

Third, this study excluded indirect and intangible costs incurred directly from medical services [28]. There were 390 reimbursement items for KM, which was significantly fewer than those for WM, and the actual percentage of non-reimbursement items is expected to be much larger [29]. Therefore, claims data from KM may not accurately reflect all treatments administered to patients with PF.

Despite these limitations, this study had the following strengths. First, this was the first study to analyze the healthcare service among patients with PF using the 2010–2018 HIRA-NPS data. No studies have compared the healthcare status of PF annually at a national level to date. Second, this study compared the status of WM and KM within the dual healthcare system of Korea, and third, this was the first study to analyze the hospital visit rates for PF according to season. Lastly, this study provided detailed information on which type of medical institution patients preferred by stratifying the patients according to age and sex.

## Conclusions

This study analyzed the HIRA-NPS data covering nine years to investigate the healthcare use in Korea related to PF. The information obtained could be used as basic data for the development of PF prevention services through exercise programs. Moreover, the findings of this study may be used as extensive reference data for future treatment and management of patients with PF. Future research should show the difference in the cost-effectiveness of each intervention by using various evaluation tools to reduce pain and increase function in patients after treatment for PF.

## Electronic supplementary material

Below is the link to the electronic supplementary material.

**Additional file 1:** Table S1. Annual average KRW-USD exchange rate and price level of health expense. Table S2. Claims of medical usage. Table S3. Average total expenditure, total number of claims, and average change rate for each for 9 years (2010–2018). Table S4. Outpatient drug prescription for plantar fasciitis. Table S5. High-frequency care for plantar fasciitis outpatients.

**Additional file 2:** Figure S1. Drug usage trend for plantar fasciitis. (A to J indicates drug categories. See Table S3). Figure S2. Anti-inflammatory drug usage trend for plantar fasciitis in detail. A-1 to A-3 indicates drug categories in detail. (See Table S3).

## Data Availability

The datasets generated and analyzed during the current study are available in the HIRA-NPS repository upon request [http://opendata.hira.or.kr] and upon payment of a data request fee.

## List of Abbreviations

PF: Plantar fasciitis
NSAIDs: Non-steroidal anti-inflammatory drugs
WM: Western medicine
KM: Korean medicine
HIRA: Health Insurance Review and Assessment Service
