# Supplementary material for: Healthcare usage and cost for plantar fasciitis: a retrospective observational analysis of the 2010–2018 health insurance review and assessment service national patient sample data
Source: BMC Health Serv Res. 2023 May 25;23:546. doi: 10.1186/s12913-023-09443-2 (PMC10210451; doi:10.1186/s12913-023-09443-2)
Supplement: Supplementary file 1 — Additional file 1: Table S1. Annual average KRW-USD exchange rate and price level of health expense. Table S2. Claims of medical usage. Table S3. Average total expenditure, total number of claims, and average change rate for each for 9 years (2010–2018). Table S4. Outpatient drug prescription for plantar fasciitis. Table S5. High-frequency care for plantar fasciitis outpatients. [file 12913_2023_9443_MOESM1_ESM.docx]

| **Supplementary Information**  **Additional file 1:**  **Table S1. Annual average KRW-USD exchange rate and price level of health expense** | | |
| --- | --- | --- |
| Year | KRW/USD | Price level |
| 2010 | 1156.00 | 0.9345 |
| 2011 | 1107.99 | 0.9510 |
| 2012 | 1126.76 | 0.9597 |
| 2013 | 1095.04 | 0.9631 |
| 2014 | 1053.12 | 0.9699 |
| 2015 | 1131.52 | 0.9820 |
| 2016 | 1160.41 | 0.9918 |
| 2017 | 1130.48 | 1.0005 |
| 2018 | 1100.58 | 1.0000 |
| This information is available on the following site: Korean Statistical Information Service (http://kosis.kr).  The price level of health expense is adjusted as of year 2018. | | |

| **Table S2. Claims of medical usage** | | | | | | | |
| --- | --- | --- | --- | --- | --- | --- | --- |
| Category | | Claims | | | | | |
|  |  | Total (2010-2018) | | WM institution (2010-2018) | | KM institution (2010-2018) | |
|  |  | n | % | n | % | n | % |
| Type of visit | Outpatient | 227,870 | 99.88 | 156,297 | 99.83 | 71,573 | 99.98 |
|  | Inpatient | 280 | 0.12 | 263 | 0.17 | 17 | 0.02 |
| Medical institution | Tertiary western hospital/ general western hospital/ western hospital | 38,788 | 17 | 38,720 | 24.73 | 68 | 0.09 |
|  | Western medicine Clinic | 117,675 | 51.58 | 117,675 | 75.16 | - | - |
|  | Korean medicine hospital | 1,776 | 0.78 | 165 | 0.11 | 1,611 | 2.25 |
|  | Korean medicine clinic | 69,911 | 30.64 | - | - | 69,911 | 97.65 |

| **Table S3. Average total expenditure, total number of claims, and average change rate for each for 9 years (2010-2018)** | | | | | | | | | | | | |
| --- | --- | --- | --- | --- | --- | --- | --- | --- | --- | --- | --- | --- |
|  | All | | | | Western Medicine | | | | Korean Medicine | | | |
|  | Total Expense | | No. of claims | | Total Expense | | No. of claims | | Total Expense | | No. of claims | |
|  | Avr. Sum | Avr. CR* | Avr. N | Avr. CR* | Avr. Sum | Avr. CR* | Avr. N | Avr. CR* | Avr. Sum | Avr. CR* | Avr. N | Avr. CR* |
| Consultation fee | 230,736 | 18.00 | 32,293 | 15.59 | 172,806 | 17.55 | 23,813 | 15.38 | 57,930 | 19.44 | 8,480 | 16.20 |
| Injection fee | 101,548 | 20.09 | 35,994 | 15.44 | 10,847 | 11.87 | 7,922 | 8.48 | 90,701 | 21.30 | 28,071 | 17.56 |
| Physical therapy fee | 57,387 | 11.87 | 30,261 | 11.90 | 57,387 | 11.87 | 30,261 | 11.90 | - | - | - | - |
| Diagnostic radiology fee | 48,364 | 18.27 | 7,745 | 9.40 | 48,364 | 18.27 | 7,745 | 9.40 | - | - | - | - |
| Treatment/surgery fee | 18,065 | 18.56 | 1,429 | 13.98 | 17,939 | 18.71 | 1,394 | 14.23 | 126 | 0.87 | 35 | 3.94 |
| [Examination fee](https://en.dict.naver.com/#/entry/enko/e3e7dcb1f4c04c358cdf7f09b87280dc) | 8,726 | 14.87 | 2,880 | 12.52 | 8,726 | 14.87 | 2,880 | 12.52 | - | - | - | - |
| Hospitalization fee | 8,718 | (0.36) | 180 | 2.82 | 7,983 | (0.10) | 167 | 3.57 | 735 | (7.24) | 13 | (1.79) |
| Prescription fee | 3,258 | 14.22 | 2,837 | 23.99 | 1,591 | (0.91) | 831 | 2.44 | 1,666 | 39.91 | 2,006 | 43.63 |
| Others | 2,003 | 12.97 | 680 | 12.03 | 2,003 | 12.97 | 680 | 12.03 | - | - | - | - |
| Avr. Sum, 9-year total expenditure average; Avr. N, average number of claims over 9 years; Avr. CR, average exchange rate over 9 years (%)  All expenses were converted with annual average exchange rate (KRW/USD). Price level of health expense is adjusted as of year 2018. (see Table S1) | | | | | | | | | | | | |

| **Table S4. Outpatient drug prescription for plantar fasciitis** | | | | | |
| --- | --- | --- | --- | --- | --- |
| **Category** | | **ATC code** | **Total Claims*** | **9-year average expense per claim** | **9-year average expense per patient** |
| A-1 | Enzymes | M09A (Anti-inflammatory enzymes) | 46,836 | 0.92 | 1.94 |
| A-2 | NSAIDs | M01A (Antipyretic, analgesic, anti-inflammatory agents) | 84,916 | 2.95 | 6.71 |
| A-3 | AAP | N02B (Antipyretic, analgesic, anti-inflammatory agents)/N02A (Antipyretic, analgesic, anti-inflammatory agents) | 26,166 | 2.20 | 4.84 |
| B | Antispasmodic | A03A (Antispasmodic)/A03B (Antispasmodic) | 4,135 | 1.52 | 3.08 |
| C-1 | Propulsives | A02B (Drugs for peptic ulcer)/A02X (Drugs for peptic ulcer) | 48,608 | 2.82 | 6.10 |
| C-2 | Other propulsives | A03F (Other gastrointestinal drugs)/A03 (Other gastrointestinal drugs)/A07B (Antidiarrheals)  A07F (Antidiarrheals)/A09A (Other gastrointestinal drugs)/A16A (Other gastrointestinal drugs) | 23,918 | 1.35 | 2.80 |
| D | Antacids | A02A (Antacids) | 5,839 | 0.47 | 1.00 |
| E | Neuro | N05B (Anxiolytics)/N03A (Antiepileptics)/N06A (Antidepressants) | 2,072 | 0.97 | 2.13 |
| F | Muscle relaxants | M03B (Skeletal muscle relaxant) | 21,625 | 1.56 | 2.97 |
| H | Hormones | G03D (Follicle-stimulating hormones and luteinizing hormones)/G01A (Pharmaceutical preparations for urogenital organs (including prophylaxis for sexually transmitted diseases))  H02A (Adrenocortical hormones) | 6,119 | 0.40 | 0.77 |
| I | Antibiotics | J01D (Acting mainly on gram-negative bacteria)/J01X (Antiprotozoal)  J01C (Acting mainly on gram-positive and negative bacteria)/J02A (Other chemotherapy)  J01A (Acting mainly on gram-positive bacteria, gram-negative bacteria, rickettsia, and chlamydia)/J02 (Other chemotherapy)  P01A (Antiprotozoal) | 469 | 4.43 | 6.60 |
| J | Circulatory system | C05CX (Other drugs for the circulatory system)/C04AX (Anticoagulant)/C03A (Diuretic) | 2,963 | 5.35 | 10.77 |
| *Among a total of 228,150 claim cases over a 9-year period, the number of cases with records of outpatient drug prescription was 90,651 (39.73%). When converted to patient unit by accounting for duplicate visits by the same patient, 60,079 patients visited a hospital for plantar fasciitis over a 9-year period, of which 40,651 patients (67.66%) received outpatient drug prescription. | | | | | |

| **Table S5. High-frequency care for plantar fasciitis outpatients** | | | | |
| --- | --- | --- | --- | --- |
|  | | Western medicine | | |
|  |  | Total claims | 9-year average expense per claim | 9-year average expense per patient |
| Examination | | 164,354 | 9.28 | 31.78 |
| Physical therapy | Heat therapy | 163,659 | 0.73 | 4.57 |
|  | Electrotherapy | 72,329 | 3.19 | 10.85 |
|  | Laser therapy | 21,462 | 5.07 | 14.96 |
|  | Iontophoresis | 11,824 | 4.18 | 12.29 |
|  | Cold therapy | 3,800 | 0.87 | 2.69 |
|  | Traction therapy | 887 | 5.87 | 19.34 |
| Joint, nerve, tendon, muscle, and subcutaneous injection | | 34,686 | 3.70 | 7.65 |
| Intravenous injection | | 1,128 | 2.84 | 8.29 |
| Radiological examination | | 54,037 | 6.38 | 11.58 |
| Blood cell/plasma composition | | 8,877 | 1.06 | 6.92 |
| Other tests | | 9,050 | 2.45 | 20.35 |
| Drug management fee | | 37,429 | 0.13 | 0.28 |
| Simple | | 769 | 6.35 | 15.46 |
| Splint | | 597 | 21.85 | 26.46 |
| Emergency medical management fee, healthcare quality evaluation grant, and emergency patient triage and screening fee | | 9,732 | 0.54 | 2.49 |
| Others | | 2,399 | 4.41 | 11.04 |
|  | | Korean medicine | | |
|  |  | Total claims | 9-year average expense per claim | 9-year average expense per patient |
| Acupuncture | | 153,774 | 3.74 | 39.16 |
| Cupping (dry cupping) | | 15,695 | 3.59 | 15.41 |
| Cupping (wet cupping) | | 11,867 | 6.00 | 16.65 |
| Moxibustion (indirect moxibustion) | | 17,762 | 2.40 | 10.12 |
| Moxibustion (direct moxibustion) | | 2,541 | 5.79 | 23.40 |
| Hot and cold meridian massage therapy | | 36,524 | 0.87 | 3.46 |
| Dispensing fee | | 7,684 | 0.44 | 1.92 |
| Examination fee | | 84,520 | 6.38 | 36.50 |
| KM testing | | 198 | 3.98 | 4.56 |
