# Supplementary material for: Healthcare usage and cost for plantar fasciitis: a retrospective observational analysis of the 2010–2018 health insurance review and assessment service national patient sample data
Source: BMC Health Serv Res. 2023 May 25;23:546. doi: 10.1186/s12913-023-09443-2 (PMC10210451; doi:10.1186/s12913-023-09443-2)
Supplement: Supplementary file 2 — Additional file 2: Figure S1. Drug usage trend for plantar fasciitis. (A to J indicates drug categories. See Table S3). Figure S2. Anti-inflammatory drug usage trend for plantar fasciitis in detail. A-1 to A-3 indicates drug categories in detail. (See Table S3). [file 12913_2023_9443_MOESM2_ESM.docx]

**Supplementary Information**


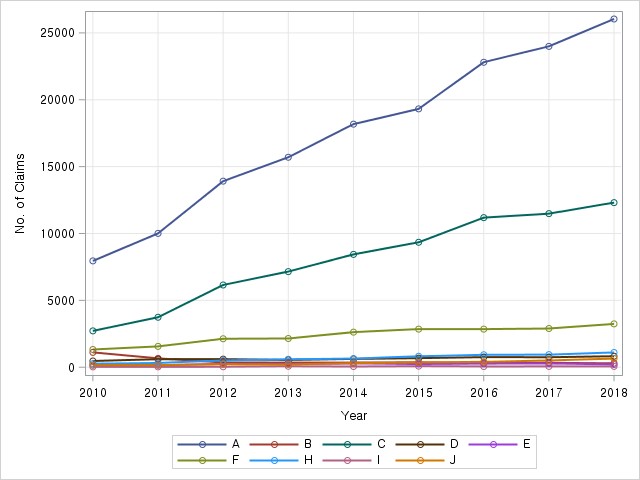


**Figure S1.** Drug usage trend for plantar fasciitis. (A to J indicates drug categories. See Table S3)


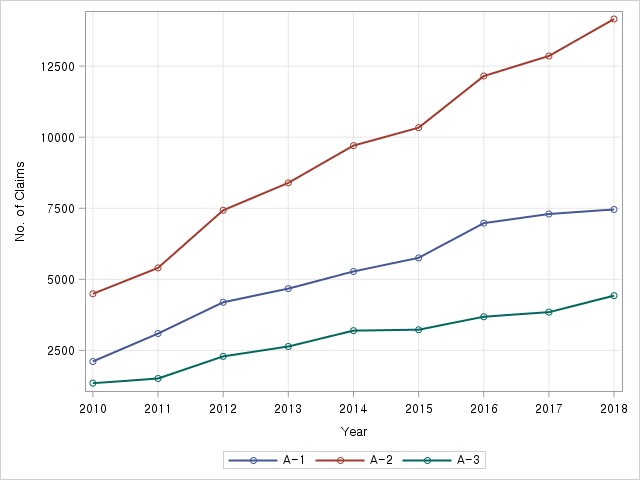


Figure S2 Anti-inflammatory drug usage trend for plantar fasciitis in detail. A-1 to A-3 indicates drug categories in detail. (See Table S3)
